# Detecting Bacterial–Human Lateral Gene Transfer in Chronic Lymphocytic Leukemia

**DOI:** 10.3390/ijms23031094

**Published:** 2022-01-20

**Authors:** Ekaterina Akimova, Franz Josef Gassner, Richard Greil, Nadja Zaborsky, Roland Geisberger

**Affiliations:** 1 Department of Internal Medicine III with Haematology, Medical Oncology, Haemostaseology, Infectiology and Rheumatology, Oncologic Center, Salzburg Cancer Research Institute—Laboratory for Immunological and Molecular Cancer Research (SCRI-LIMCR), Paracelsus Medical University, Cancer Cluster Salzburg, 5020 Salzburg, Austria; e.akimova@salk.at (E.A.); f.gassner@salk.at (F.J.G.); r.greil@salk.at (R.G.); n.zaborsky@salk.at (N.Z.); 2 Department of Biosciences, Paris Lodron University of Salzburg, 5020 Salzburg, Austria

**Keywords:** CLL, lateral gene transfer (LGT), bacterial integrations

## Abstract

Chronic lymphocytic leukemia (CLL) is a very common and mostly incurable B-cell malignancy. Recent studies revealed high interpatient mutational heterogeneity and worsened therapy response and survival of patients with complex genomic aberrations. In line with this, a better understanding of the underlying mechanisms of specific genetic aberrations would reveal new prognostic factors and possible therapeutic targets. It is known that chromosomal rearrangements including DNA insertions often play a role during carcinogenesis. Recently it was reported that bacteria (microbiome)–human lateral gene transfer occurs in somatic cells and is enriched in cancer samples. To further investigate this mechanism in CLL, we analyzed paired-end RNA sequencing data of 45 CLL patients and 9 healthy donors, in which we particularly searched for bacterial DNA integrations into the human somatic genome. Applying the Burrows–Wheeler aligner (BWA) first on a human genome and then on bacterial genome references, we differentiated between sequencing reads mapping to the human genome, to the microbiome or to bacterial integrations into the human genome. Our results indicate that CLL samples featured bacterial DNA integrations more frequently (approx. two-fold) compared to normal samples, which corroborates the latest findings in other cancer entities. Moreover, we determined common integration sites and recurrent integrated bacterial transcripts. Finally, we investigated the contribution of bacterial integrations to oncogenesis and disease progression.

## 1. Introduction

Chronic lymphocytic leukemia (CLL) is a very common B-cell malignancy that mostly affects elderly people [1]. CLL patients show a vastly heterogeneous mutational profile, comprising a high number of passenger and driver mutations, which forward clonal evolution and the course of disease [2,3]. Several somatic mutations and chromosomal aberrations were identified as prognostic factors, and are now being used in clinics for therapy choice. For example, *IGHV* mutated patients have a better prognosis and respond better to fludarabine-cyclophosphamide-rituximab (FCR) chemoimmunotherapy, which leads to prolonged progression-free survival [4,5]. Moreover, mutations in *TP53* and *ATM* genes or complex chromosomal alterations such as del17q, del11q and tri12 correspond with worsened prognosis and survival [2,3]. Moreover, a complex karyotype (≥3 distinct chromosomal abnormalities) leads to impaired therapy response [6]. Alongside deletions, duplications and translocations, integrations of foreign DNA represent another type of genomic aberration that can influence carcinogenesis. Various transposon types are well described and are known to contribute to oncogenesis once they become mobile. In the context of leukemia, *Alu* and SINE-VNTR-Alu (SVA) retrotransposon insertions play a major role [7]. Furthermore, integrations of mitochondrial DNA into the nuclear genome represent a recurrent genetic alteration in cancer cells [8]. The causative effects of some viral integrations on cancer development have been known for two decades, and investigated intensively since. The most prominent examples are human papillomavirus (HPV) integrations in cervical malignancies [9] and hepatitis B virus (HBV) integrations in hepatocellular carcinoma [10]. Often these viral integrations occur in oncogenes or tumor-suppressor genes, resulting in increased cell proliferation and downregulation of apoptotic pathways [11]. A recent survey assesses that over two million cancer cases per year are infection-associated (data for 2018) [12]. While most of the described diseases are caused by viruses including HPV and HBV, over a third of reported cases are attributed to infections with *Helicobacter pylori*. However, the known role of bacteria during tumorigenesis was restricted to their inflammatory properties and to the production of DNA-damaging agents [13,14]. A paper published by Riley et al. revealed that somatic bacterial integrations into the human genome are detectable in several cancer types, suggesting bacteria-to-human lateral gene transfer (LGT) by pathologic or commensal bacteria to be a novel mechanism possibly contributing to cancerogenesis [15]. These data prompted us to investigate bacteria-to-human LGT in a cohort of clinically well-characterized CLL patients and to examine whether the magnitude of LGT associates with prognostically relevant parameters or treatment outcome.

## 2. Results

### 2.1. Detecting Bacterial Integrations into the Human Genome in CLL Patients

**Figure 1:**
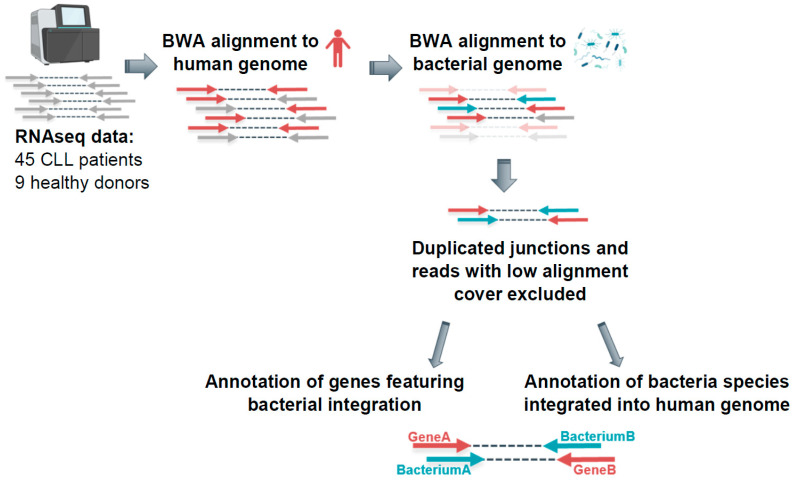
Detecting bacterial integrations into the human genome in CLL patients—analysis workflow. RNAseq data were aligned to the human genome (hg19) and subsequently to bacterial genome references via BWA. The reads supporting LGT were defined as reads featuring only one mate mapping exclusively to human genome and only one mate mapping exclusively to bacterial genome.

Based on a recent study that described bacterial–human lateral gene transfer in several cancer entities [15], we customized the analysis workflow to examine the bacterial integration rate in CLL patients. The paired-end RNA sequencing data from 45 CLL samples and 27 healthy B-cell samples (naïve, non-class-switched and class-switched memory B-cell subsets from 9 healthy donors) were processed. First, the RNA sequencing reads were aligned to the human genome using the BWA method (Figure 1).

An output for each read pair indicated if both mates, only the first, or only the second mate mapped to the human genome. For further analysis, the read pairs mapping with only one mate to the human genome were selected and aligned to bacterial genome references. The reads which mapped with one mate to human and with another mate to bacterial reference were defined as reads potentially supporting bacterial–human lateral gene transfer. In the next filtering step, we excluded all reads with <90% alignment coverage and low-complexity reads containing long poly-A/-T/-G/-C sequences. Furthermore, we excluded duplicated junctions, which were defined by the genomic position of the integration and the genomic position on respective bacterial reference of the integrated fragment, to rule out possible technical artefacts.

### 2.2. Reads Supporting Bacterial–Human LGT were Enriched in CLL versus Non-Leukemic B Cells

**Figure 2:**
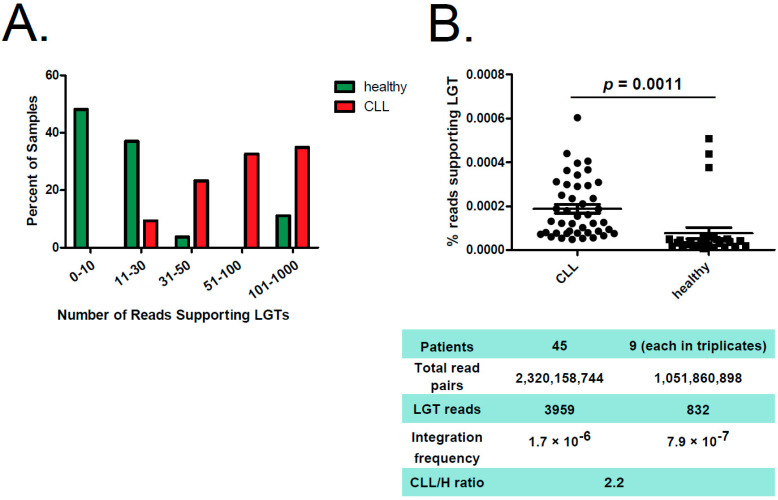
Reads supporting bacterial–human LGT were enriched in CLL versus non-leukemic B cells. (**A**) Distribution of reads supporting LGT in CLL samples (red) and in healthy B cells (green). The graph shows the percentage of samples featuring indicated number of reads supporting LGT. (**B**) The figure displays a comparison between CLL samples from CLL patients and B-cell samples from healthy (H) donors, whereas percentage of reads supporting bacterial–human lateral gene transfer is shown. Median is indicated. Significance was calculated using unpaired two-tailed *t*-test. Below, key information is summarized for both groups.

In total, we analyzed 2,320,158,744 read pairs originating from CLL samples and 1,051,860,898 from healthy B-cell samples. Although we could detect at least three LGT events per healthy sample, each CLL sample featured 23 or more unique LGT events. Most of the CLL patients had >50 reads supporting LGT (Figure 2A, Supporting Figure S1, Supplementary Table S1). Furthermore, the number of reads supporting LGT was normalized to the total read number. This revealed that CLL patients had 2.2-times higher frequency of bacterial integrations into the human genome compared to healthy donors (Figure 2B; *p*-value = 0.0011). Notably, all three outliers in the healthy cohort with elevated numbers of LGT events originated from class-switched B cells. To corroborate these results, we accessed and analyzed additional RNAseq data from in-vitro-EBV-transformed B cells (*n* = 15), naïve B cells (*n* = 10) as well as memory B cells (*n* = 3). Thereby, we could confirm low-level LGT in naïve and EBV-transformed B cells and a high LGT level in memory B cells (Supporting Figure S2, Supplementary Table S2).

### 2.3. Bacterial Integrations Occurred Recurrently in Several Genes

**Figure 3:**
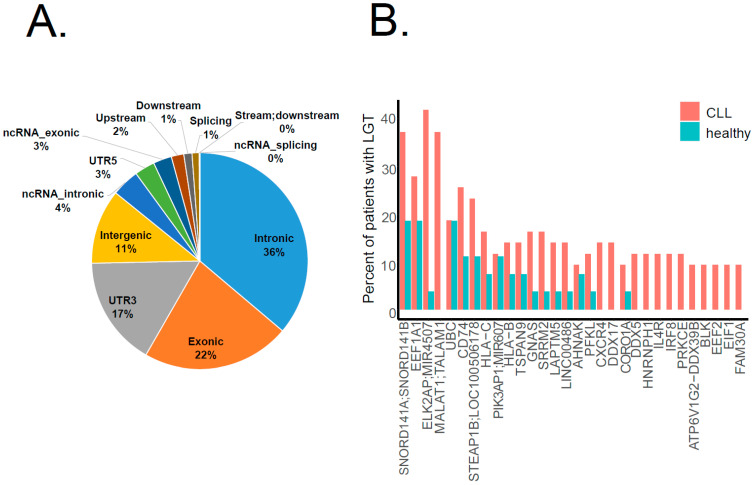
Genomic regions affected by bacterial–human LGT. (**A**) The pie chart shows the distribution of LGT events in the CLL cohort among different gene coding structures: most of them (36%) occurred in intronic regions. (**B**) The bar plot shows the top 30 genes affected by LGT. The Y-axis indicates the percentage of patients in each cohort (CLL in pink, healthy in blue) featuring a bacterial integration in the respective gene.

According to gene coding structure annotation, the majority of bacterial integration events (36%) occurred in intronic regions, 22% in exonic regions and 17% in UTR3′ (Figure 3A). While we detected bacterial integrations throughout the human genome, several genes were identified as hot spots for LGT, which recurrently appeared in a number of patients. Figure 3B summarizes the top 30 genes with a bacterial integration in both CLL and healthy cohorts. Interestingly, three of the four top genes (*SNORD141A/B*, *MIR4507* and *MALAT1*) are non-coding RNA transcripts, which are reported to be disease associated, among others in lung adenocarcinoma [16] and B-cell malignancies [17]. Notably, LGT in *CD74* (here, the top sixth gene) was also described for stomach adenocarcinomas [15]. CD74, or MHC class II-associated invariant chain, plays an important role in antigen presentation. It also modulates several survival pathways, including the NF-κB pathway, which are associated with oncogenesis [18,19]. Intriguingly, the top 10 genes featuring LGT in our analysis also comprised *HLA-B* and *HLA-C* genes, which encode two of three main types of class I major histocompatibility complex (MHCI), whereas the *HLA-A* gene is known to be affected by retrotransposons, particularly by *SVA* integration, in the leukemic context [7].

### 2.4. Some Bacteria Genera Appear to Integrate into Human Genome with Higher Likelihood

**Figure 4:**
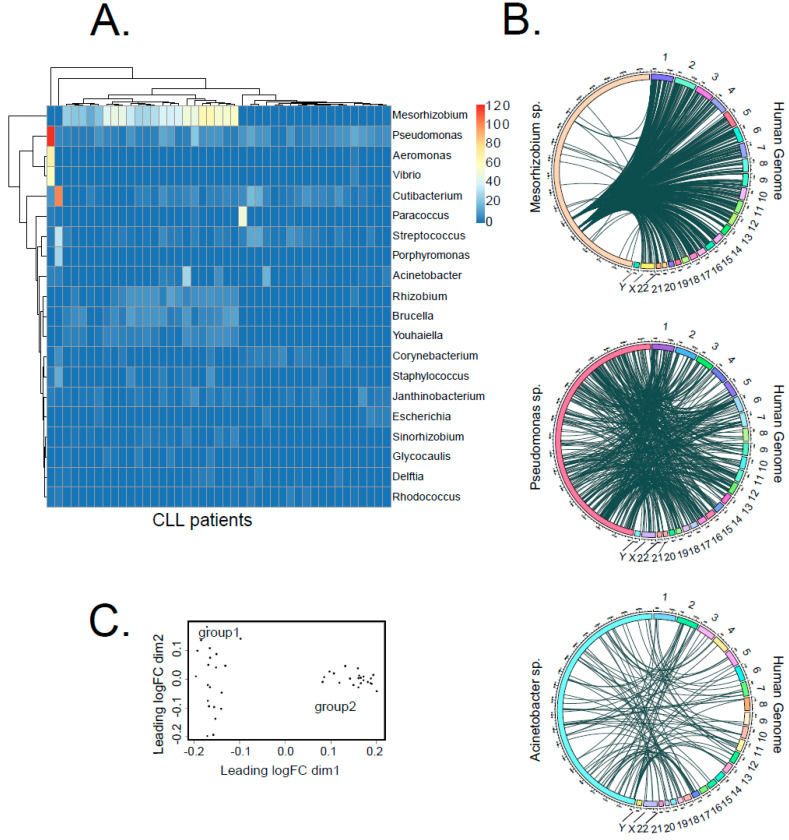
Distribution of bacterial species contributing to LGT in CLL. (**A**) The heat map shows the number of bacterial transcripts integrated into human genome from the 20 most abundant bacteria genera for each CLL sample. In total, we detected 216 different bacteria genera across the entire dataset. (**B**) Circos plots illustrate the integration sites of three selected bacteria genera (*Pseudomonas* sp., *Mesorhizobium* sp. and *Acinetobacter* sp.) into the human genome. (**C**) MDS analysis of LGT event counts, using a matrix that lists the number of LGT events for each bacteria genus per patient.

Throughout the data from the CLL cohort, we identified 216 bacteria genera integrated into the human genome at distinct locations (Supporting Figure S3A, Supplementary Table S1), compared to 113 genera for healthy controls (Supporting Figure S3B, Supplementary Table S1). However, there was a large variance in the abundancies of different genera (Figure 4A). Whereas *Pseudomonas* sp., the second most common genus detected, was present in almost all analyzed samples with mostly comparable counts, *Mesorhizobium* sp., the most common genus detected, was highly abundant in one group of patients (22 patients) and absent in the others. Our analysis showed no specific integration sites preferably used by specific bacteria. To visualize the insertion map, we generated circos plots, which exemplarily show the integrations of three bacteria genera, *Pseudomonas* sp., *Mesorhizobium* sp. and *Acinetobacter* sp., which are distributed across the whole human genome (Figure 4B).

Interestingly, *Pseudomonas* was also reported to be the most prevalent integrated taxonomic unit in stomach adenocarcinoma [15]. Infection with *Pseudomonas aeruginosa* was further reported to stimulate gastric cancer development in animal experiments [20]. Moreover, integrations from the genus *Acinetobacter*, which had the ninth highest abundance in our cohort, were determined as the most frequent in acute myeloid leukemia by Riley et al. [15]. Until now, there was no particular evidence of DNA integrations from the genus *Mesorhizobium* into the human genome or of its role during carcinogenesis. Nevertheless, it was described that *Mesorhizobium* sp. was increased in the microbiome of patients suffering from benign biliary pathology, although it was barely present in a healthy human bile duct microbiome [21].

Furthermore, the multidimensional scaling analysis (MDS) of genus-specific bacterial integration counts divided CLL patients into two groups (group 1, *n* = 21 and group 2, *n* = 22, Figure 4C). This classification nicely correlated with a general frequency of LGT, with group 2 showing 2.8 times more LGT events than group 1 (Figure 4C, Supporting Figure S4).

### 2.5. Correlation of LGT Events with Clinically Relevant Parameters

**Figure 5:**
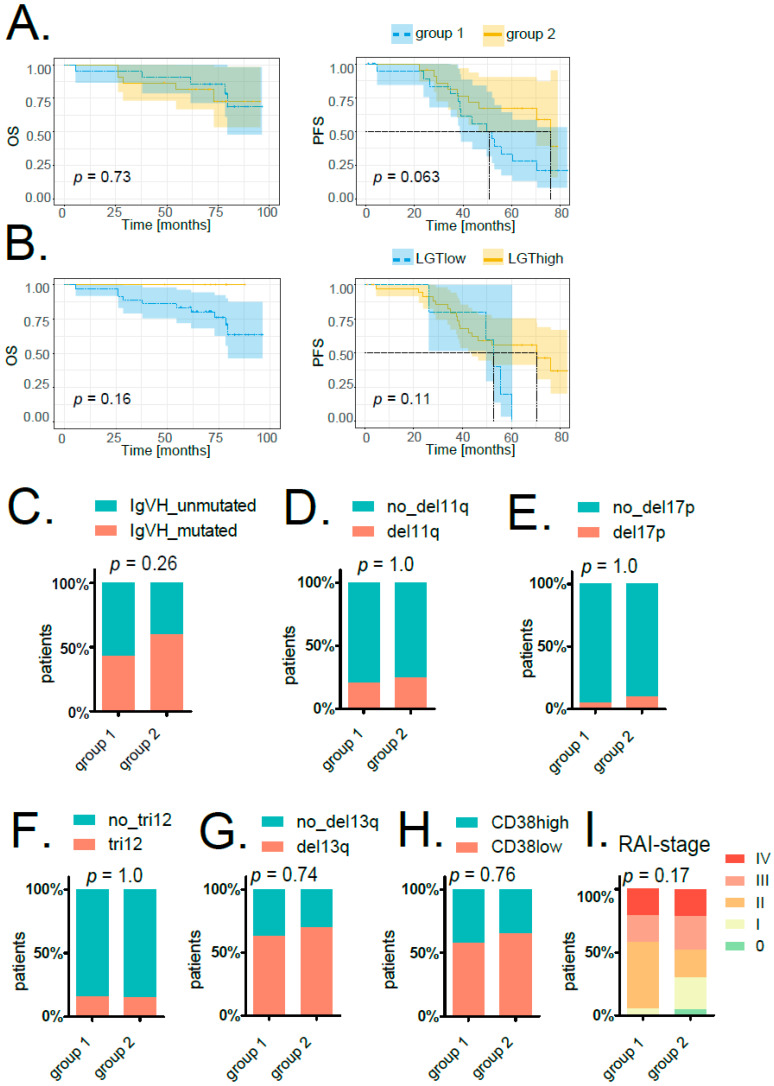
Correlation of LGT events with clinically relevant parameters. (**A**) Kaplan–Meier overall survival (OS, left) and progression-free survival (PFS, right) curves comparing patients grouped according to MDS analysis of LGT events (groups 1 and 2). (**B**) Kaplan–Meier overall survival (OS, left) and progression-free survival (PFS, right) curves comparing patients with higher (LGThigh, in yellow) and lower (LGTlow, in blue) amounts of bacterial integrations (cutoffs calculated by Cox regression analysis). For all survival plots’ *p*-values, confidence intervals as well as horizontal and vertical median survival lines are indicated. The stacked bar plots show the percentage of patients in group 1 and group 2 either featuring *IGVH* mutation (**C**), del11q (**D**), del17q (**E**), tri12 (**F**), del13q (**G**) or not. (**H**) The stacked bar plot indicates the percentage of patients in group 1 and group 2 with either high or low CD38. For (**C**–**H**), significances were calculated with a two-sided Fisher’s exact test. (**I**) The stacked bar plot displays the percentage of patients in group 1 and group 2 in different stages according to the assigned RAI index. Significance was calculated with Pearson’s chi-squared test.

Finally, we examined if patient classification based on LGT events corresponded to clinically relevant parameters. We compared progression-free survival (PFS) and overall survival (OS) time upon treatment between LGT group 1 and group 2 patients (Figure 5A) and between patients grouped according to the number of LGT events (cutoffs were calculated by Cox regression analysis; for PFS: LGTlow < 5.82947 × 10^–5^% LGT events, *n* = 5; LGThigh > 5.82947 × 10^–5^% LGT events, *n* = 38; for OS: LGTlow < 0.000309331% LGT events, *n* = 36; LGThigh > 0.000309031% LGT events, *n* = 7; Figure 5B). We did not observe any significant differences in survival between the respective groups, although there was a trend towards prolonged PFS in group 2 patients (Figure 5A). Associating LGT events with prognostically relevant parameters such as IGVH mutation status, RAI staging or common cytogenetic aberrations determined by FISH also did not reveal any significant correlations (Figure 5C–I, Supporting Figure S5).

## 3. Discussion

Lateral (or horizontal) gene transfer describes DNA integrations from one organism into the genome of another. LGT is considered an evolutionary mechanism, and is especially important for bacteria to acquire new properties not only from other related bacteria, but also from eukaryotes, including humans [22,23]. Lateral gene transfer in the opposite direction as an evolutionarily relevant event is evident as well, considering that many genes within the human genome seem to originate from bacterial genomes [24,25]. On the other hand, bacterial integrations into somatic genomes may, on an individual level, lead to gene recoding or to the overexpression of oncogenes, thus altering cellular homeostasis and causing disease development. Accordingly, a study by Riley et al. confirmed the presence of LGT events in various cancers [15]. In our study, we for the first time analyzed the load and nature of bacterial integrations in chronic lymphocytic leukemia patients using RNA sequencing data and stratified patients according to LGT events and clinical parameters. Although the cohort of 45 patients was rather small, the patients were clinically well characterized and allowed a thorough correlation of LGT grouping with prognostic and predictive factors. Intriguingly, although the overall frequency of LGT events was fairly low, we detected a more than two times higher integration rate in CLL samples compared to healthy controls. Of note, a high integration rate was also detected in class-switched memory B cells, showing that LGT may increase with the lifespan of a cell and with high activity of the DNA repair machinery required for immunoglobulin class-switch recombination. In this regard, CLL patients often feature a high number and wide range of different mutations, among others, complex chromosomal aberrations [3], possibly resulting from an intrinsically lower ability to precisely repair DNA damage [26]. Likely, the higher propensity to acquire DNA aberrations may also contribute to the integration of foreign DNA into the somatic genome of tumor cells. While in some other cancer types (e.g., in cancers arising in the gastrointestinal tract) the interaction with the microbiome is certainly higher than in CLL, the contact of malignant B cells with bacterial DNA could occur in the blood due to the presence of minute amounts of cell-free DNA originating from current or past infections [27], or from the microbiome [28]. In addition, bacterial DNA could be transported to lymphoid tissue—whereupon it is available for CLL cells—during constant processing and presentation of microbiome-derived antigens by innate immune cells [29]. Although our study did not reveal any robust association of LGT events with clinically relevant prognosticators such as IGVH mutation status, chromosomal aberrations or RAI staging, we noticed a trend towards differential PFS upon treatment in patients grouped according to LGT events. The fact that LGT was also detected in healthy cells could point to LGT as an inevitable by-product of DNA repair rather than a strong cancer-driving process. However, although it is conceivable that in most cases LGT has no effect on cell homeostasis, it may contribute to the clonal evolution of cancer cells in individual cases.

In summary, we were able to show that bacteria-to-human LGT frequently occurs in CLL. However, as LGT likely has only a subtle impact on disease pathogenesis, its contribution to disease development, progression and treatment outcome remains to be analyzed in the longitudinal tracking of large patient cohorts.

## 4. Materials and Methods

The LGT detection method was based on a workflow published by Riley et al. [15]. Paired-end RNA sequencing data from 45 untreated CLL patients sampled prior treatment with lenalidomide in combination with fludarabine and rituximab (AGMT-REVLIRIT trial, ClinicalTrials.gov Identifier: NCT00738829 and NCT01703364) [30] and 9 healthy B-cell donors (each in triplicates, 27 samples; downloaded from the European Genome-phenome Archive: EGAS00001000374) [31] were aligned to the human genome (bwa mem hg19; bwa v0.7.12-r1039). Reads that mapped with only one mate to hg19 were extracted (SAMtools view -h -f 4 -F 8 -F 2048, SAMtools view -h -f 8 -F 4 -F 2048; SAMtools merge; SAMtools v1.10), reformatted to FASTQ (Picard SamToFastq; Picard v2.21.9) and realigned to bacterial genome references (RefSeq, NCBI) using the same parameters. The reads supporting LGT were defined as reads featuring only one mate mapping exclusively to the human genome and only one mate mapping exclusively to the bacterial genome. Thus, the integration junction sequence lies in-between the two mates (within the unsequenced insert) of the same RNA fragment. The post-alignment filtering and analysis were performed on a Linux Redhat system using custom Bash and R (v4.0.4) scripting. All reads with <90% alignment coverage and low-complexity reads with long poly-A/-T/-G/-C sequences were excluded. Additionally, the integration sites were intersected with RepeatMasker file, available online: https://genome.ucsc.edu/cgi-bin/hgTables (accessed 15 December 2021) using Bedtools v2.26.0 in order to identify integration sites in low-complexity regions. Moreover, duplicated integrations (integrations of the same bacterial transcript region at the same genomic position) were excluded from further analysis. For gene and gene structure annotation, the ANNOVAR software tool (Version: 2017-07-17) was applied. The following R packages were used: dplyr, ggplot2, circlize, survival, survminer, limma, pheatmap.

## Data Availability

Customized bioinformatics scripts are available upon request.

## Supplementary Materials

The following are available online at https://www.mdpi.com/article/10.3390/ijms23031094/s1.
