# Supplementary material for: Detecting Bacterial–Human Lateral Gene Transfer in Chronic Lymphocytic Leukemia
Source: Int J Mol Sci. 2022 Jan 20;23(3):1094. doi: 10.3390/ijms23031094 (PMC8835664; doi:10.3390/ijms23031094)
Supplement: Supplementary file 1 [file ijms-23-01094-s001.zip › Supporting_figure_legends.pdf]

Supporting Figure S1. Distribution of reads supporting LGT in CLL samples (red) and in healthy B cells (green). The graph shows the percentage of samples featuring indicated percentage of reads supporting LGT.

Supporting Figure S2. The graph shows the percentage of reads supporting LGT in different non-leukemic B cell entities and CLL. The median is indicated as bar.

Supporting Figure S3. Distribution of bacterial species contributing to LGT in CLL (A) and in healthy samples (B). The heat maps show the number of bacterial transcripts integrated into human genome from all detected bacteria genera for each sample.

Supporting Figure S4. The dot plot compares between group 1 and group 2, which were defined by MDS analysis (Figure 4C), whereas percentage of reads supporting bacterial-human lateral gene transfer is shown. Median is indicated. Significance was calculated using unpaired two-tailed t test.

Supporting Figure S5. The dot plots indicate percentage of reads supporting LGT in patients either featuring del17q (A), del11q (B), del13q (C), tri12 (D), IGVH mutation (F) or not or having either high or low CD38 (E). (G) The box plot displays the amount of reads supporting LGT in CLL patients in different stages according to assigned RAI index. For A-G: median is indicated; significances were calculated using unpaired two-tailed t test.

Supplementary Table S1. List of detected LGT events for CLL and healthy cohorts. The table summarizes alignment scores, sequences and annotates identified bacteria.

Supplementary Table S2. List of detected LGT events for naïve, memory B cells and B cells immortalized in vitro with EBV. Data accessed from bioprojects PRJEB3366, PRJNA731107, PRJNA762548 and PRJNA626563.
