# Supplementary figures and images for: Detecting Bacterial–Human Lateral Gene Transfer in Chronic Lymphocytic Leukemia
Source: Int J Mol Sci. 2022 Jan 20;23(3):1094. doi: 10.3390/ijms23031094 (PMC8835664; doi:10.3390/ijms23031094)

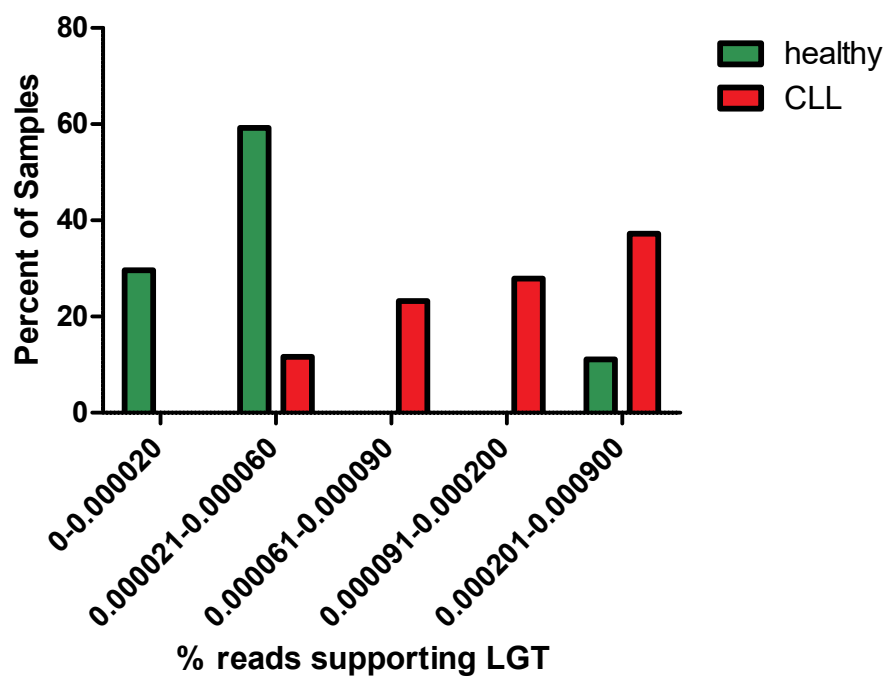

Supporting Figure S1.

Supplement: Supplementary file 1 [file ijms-23-01094-s001.zip › Figure S1.pdf]

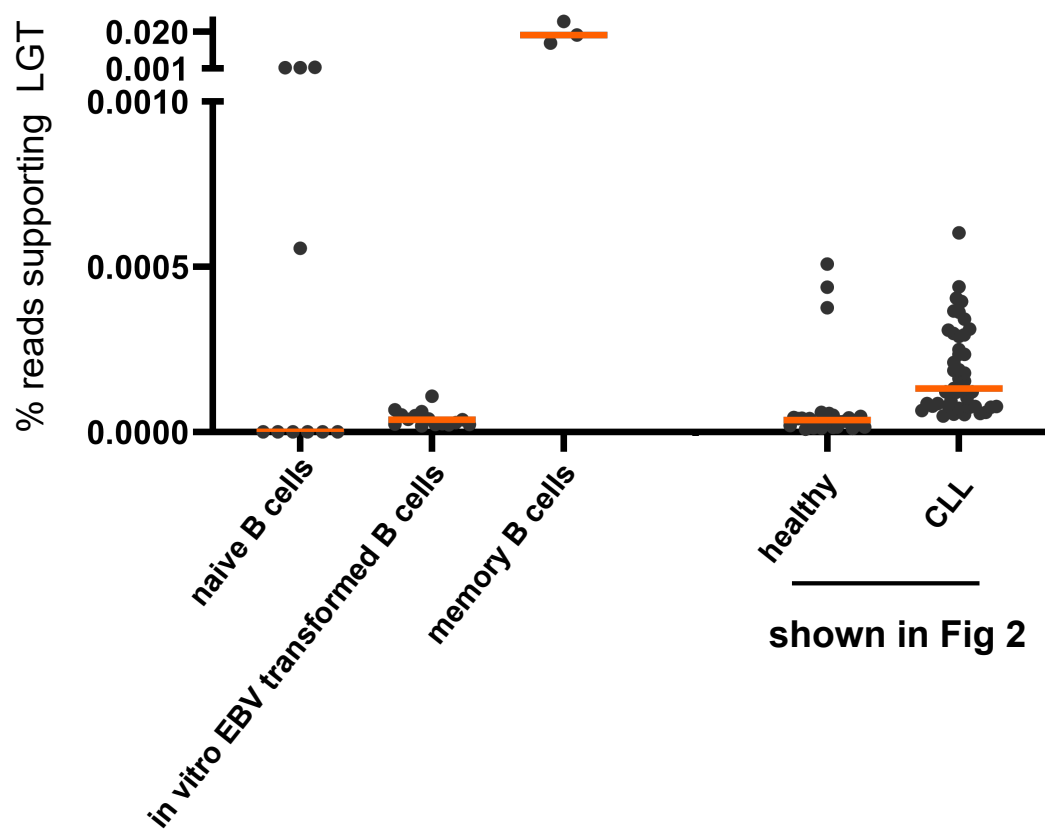

**Supporting Figure S2**

Supplement: Supplementary file 1 [file ijms-23-01094-s001.zip › Figure S2.pdf]

**A.**

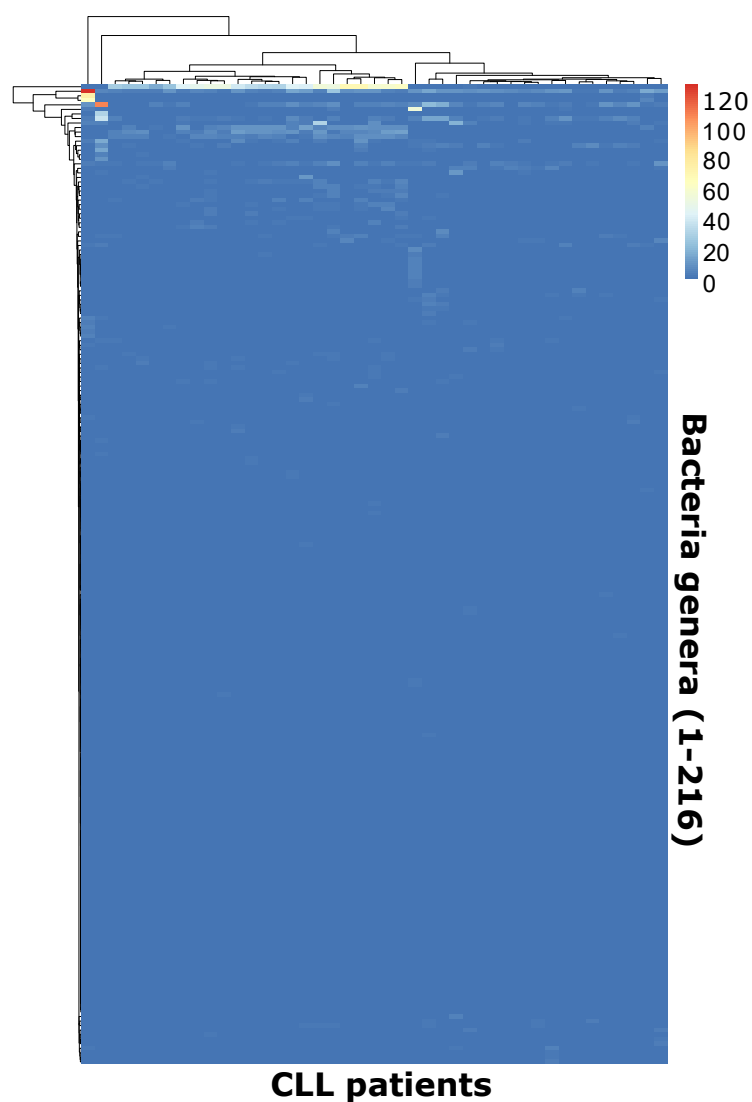

**B.**

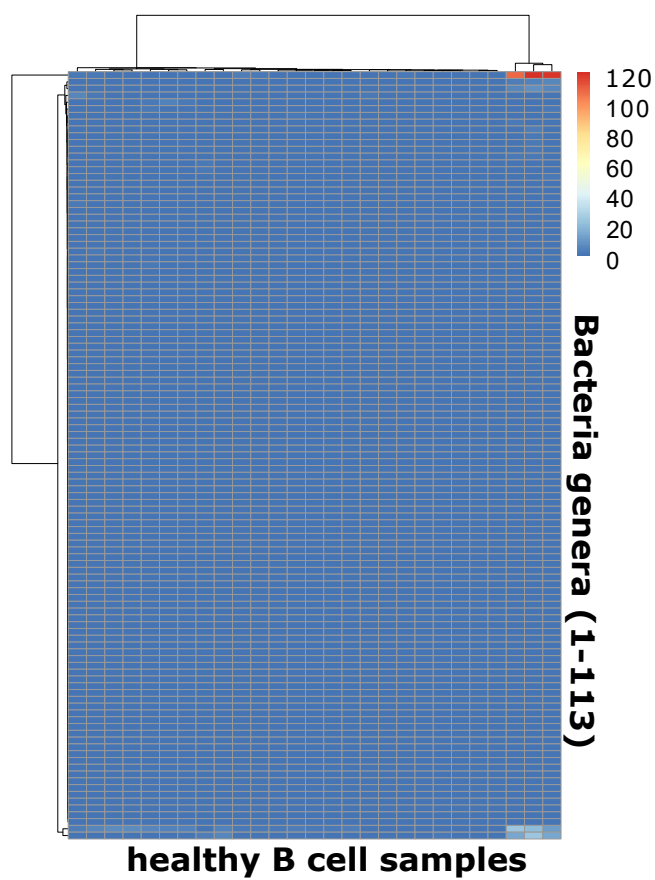

**Supporting Figure S3**

Supplement: Supplementary file 1 [file ijms-23-01094-s001.zip › Figure S3.pdf]

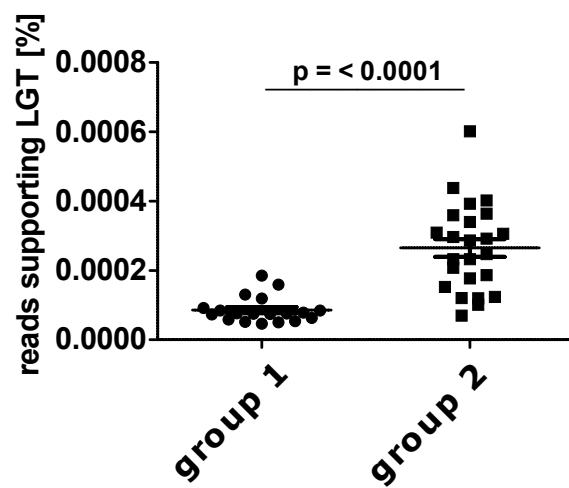

**Supporting Figure S4**

Supplement: Supplementary file 1 [file ijms-23-01094-s001.zip › Figure S4.pdf]

A.

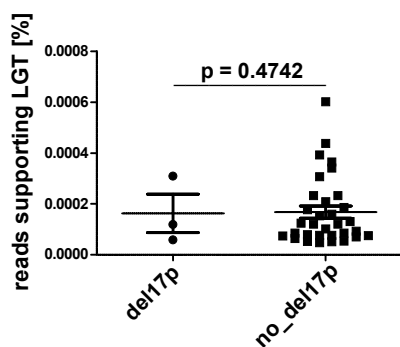

B.

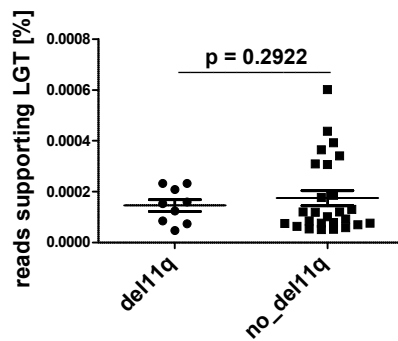

C.

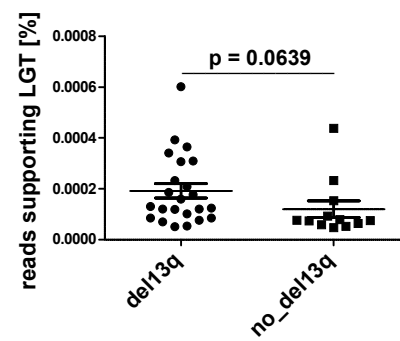

D.

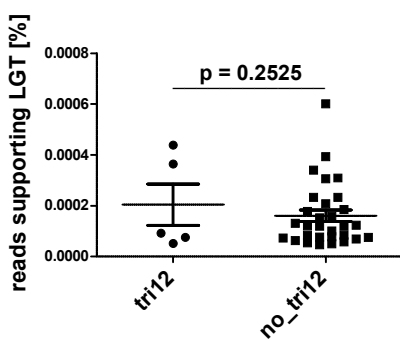

E.

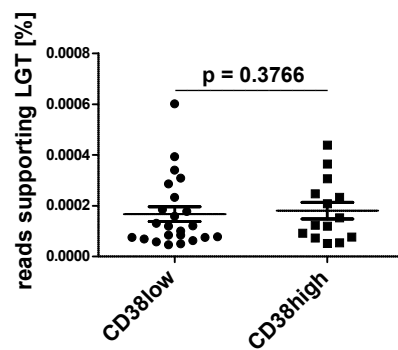

F.

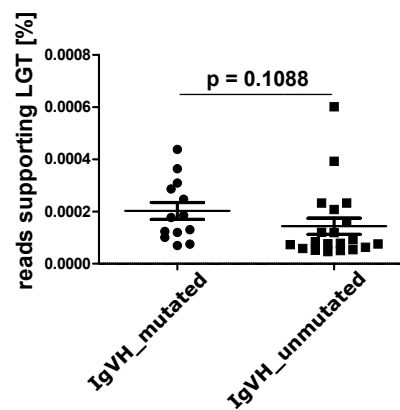

G.

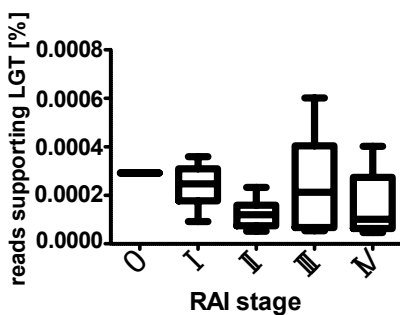

Supporting Figure S5

Supplement: Supplementary file 1 [file ijms-23-01094-s001.zip › Figure S5.pdf]
